# Change of Name

**Published:** 1872-09

**Authors:** 


### CHANGE OF NAME.

A desire has been expressed by a number of our readers, that as our journal is, to a large degree, the representative organ of the profession South, its name should be changed in order to accord with its character in this respect. In view, therefore, of these requests we invite our Associates and readers to suggest a name for it which shall express more fully its scope and design. Their suggestions will then be submitted to a committee of our Associates, and the name changed (January next) according to their recommendation. Friends, let us hear from you.

